# A follow-up study to assess the determinants and consequences of physical activity in pregnant women of Cuenca, Spain

**DOI:** 10.1186/s12889-016-3130-x

**Published:** 2016-05-25

**Authors:** Raquel Poyatos-León, Gema Sanabria-Martínez, Jorge Cañete García-Prieto, Celia Álvarez-Bueno, Diana P. Pozuelo-Carrascosa, Iván Cavero-Redondo, Antonio García-Hermoso, Sagrario Gómez-Cantarino, Miriam Garrido-Miguel, Vicente Martínez-Vizcaíno

**Affiliations:** Virgen de la Luz Hospital, Cuenca, Spain; Universidad de Castilla-La Mancha, Social and Health Research Center, Cuenca, Spain; Laboratorio de Ciencias de la Actividad Física, el Deporte y la Salud, Facultad de Ciencias Médicas, Universidad de Santigo de Chile, USACH, Santiago, Chile; Universidad de Castilla-La Mancha, Facultad de Enfermería, Toledo, Spain; Universidad Autónoma de Chile, Facultad de Ciencias de la Salud, Talca, Chile

**Keywords:** Physical activity, Pregnancy outcomes, Neonatal outcomes, Accelerometer

## Abstract

**Background:**

In recent years, the influence of physical exercise on pregnancy outcomes has been widely debated. Despite the numerous studies addressing the relationship between maternal physical activity and pregnancy outcomes, the evidence for consistent and significant impact of regular exercise during pregnancy on fetal growth remains lacking. The aims of this study were, first, to assess the level of physical activity performed throughout the pregnancy by objective (accelerometer) and self-reported (questionnaire) measurements, and, second, to ascertain pre-pregnancy physical activity levels, to estimate the relationship between levels of physical activity and some pregnancy and neonatal outcomes.

**Methods/design:**

This was a prospective cohort study. Participants were pregnant women (*n =* 194) aged 18 to 40 years who attended for three quarterly appointments for pregnancy ultrasound scans at the Virgen de la Luz Hospital in Cuenca, Spain. All participants provided written informed consents to participate in the study. Physical activity during the pregnancy follow-up was assessed by a self-reported Pregnancy Physical Activity Questionnaire and sleep log; also objectively by a GT3X accelerometer (ActiGraph). Furthermore, pregnancy symptoms inventory, nutritional behavioural assessment, socio-demographic characteristics, and anthropometry and body composition were measured.

At the end of the follow up, the following main outcomes were determined: pregnancy outcomes (incidence of gestational diabetes mellitus, pre-eclampsia, pregnancy-induced hypertension, weight gain during pregnancy, type of delivery, and neonatal outcomes (gestational age, birth weight, gender, Apgar score 1 min/5 min, type of resuscitation (I/II/III/IV), and pH of umbilical cord blood). Descriptive statistics for cross-sectional data, linear mixed regression models for absolute differences in changes baseline-final measurements were used as statistical analyses.

**Discussion:**

Although the effectiveness of physical activity programmes on improving maternal and neonatal outcomes has heretofore been studied, the impact of free time physical activity during pregnancy has not been assessed using objective measures. This paper reports the design of a prospective cohort study that aims to assess the physical activity levels of pregnant women, and to estimate the relationship between those physical activity levels with maternal and neonatal outcomes.

This study could contribute to providing evidence for the formulation of recommendations for physical activity for pregnant women.

## Background

Pregnancy is a period characterised by a greater awareness of health and care. Therefore, pregnant women are more likely to make changes in their lifestyle, which may lead to healthier behaviours. The benefits of these changes affect not only the mother’s health but also the type of delivery at birth and the well-being of the new-born child. However, pregnant women are often encouraged to reduce their levels of physical activity and even to stop working because of the belief that physical activity may reduce placental circulation and, consequently, increase the risk of disorders such as miscarriages, preterm deliveries, and intrauterine growth retardation. Also, concerns have been expressed about other potentially negative effects of physical activity during pregnancy such as increase in body temperature, the risk of maternal muscle-skeletal injuries, and the diversion of maternal O2 and nutrients to skeletal muscle rather than to the fetus [1].

Research has provided new information on the positive influence of physical activity on women and their infants [2–4]. Some of the benefits identified are: the improvement of cardiovascular function, the limitation of weight gain during pregnancy, the reduction of musculoskeletal discomfort [5], the incidence of muscle cramps and edema on lower limbs [6], lower mood, decreased risk of gestational diabetes mellitus (GDM) [7, 8] and pregnancy-induced hypertension [5, 9], and, fewer complications at birth [10]. Moreover, neonatal benefits arising from maternal physical activity have been described as decrease in fat mass, higher stress tolerance and earlier neurobehavioural development [11].

Based on these findings, the current physical activity recommendations of the American College of Obstetricians and Gynaecologists (ACOG) encourage women, without growing contraindications, to be physically active throughout pregnancy and to have at least 20–30 min of moderate-intensity exercise on most, if not all, days [12]. However, this guideline does not define ‘moderate intensity’ or the specific amount of weekly energy expenditure required from physical activity. Moreover, research data have posited the necessity for incorporating strength training and muscle conditioning in pregnant women’s physical activity [13].

During pregnancy, the mother undergoes a variety of physiological and metabolic adjustments. These functional changes make it possible for the mother to sustain fetal growth while protecting her own homeostasis. Estrogen and progesterone produced by the placenta are responsible for most of the changes taking place in the maternal body. These hormones also play a crucial role in establishing an adequate maternal-fetal exchange of nutrients by way of increasing uteroplacental blood flow. Without such hormonal activity, threats to maternal well-being in terms of nutrient availability could disrupt maternal-fetal exchange with negative consequences for the fetus [14]. It is essential to ascertain the level of weekly energy expenditure required of pregnant women. Therefore, further scientific evidence for the effects of physical activity on maternal and neonatal outcomes seems required.

## Aims

The main objective of our study was to assess the physical activity levels (type, frequency and intensity) of pregnant women by using accelerometers and self-reported questionnaires measurements (pregnancy symptoms inventory and sleep log).

The secondary objectives of the study were: i) to assess pre-pregnancy physical activity levels; ii) to estimate the relationship between physical activity levels during pregnancy with pregnancy outcomes (incidence of GDM, pre-eclampsia, pregnancy-induced hypertension, weight gain during pregnancy, and type of delivery), and neonatal outcomes (gestational age, birth weight, gender, Apgar score 1 min/5 min, type of resuscitation (I/II/III/IV), and pH of umbilical cord blood.); iii) to identify barriers and facilitators that influence the performance of exercise during pregnancy; and, iv) to examine nutritional behaviours during pregnancy through YANA-C.

## Methods/design

### Study design

**Fig. 1:**
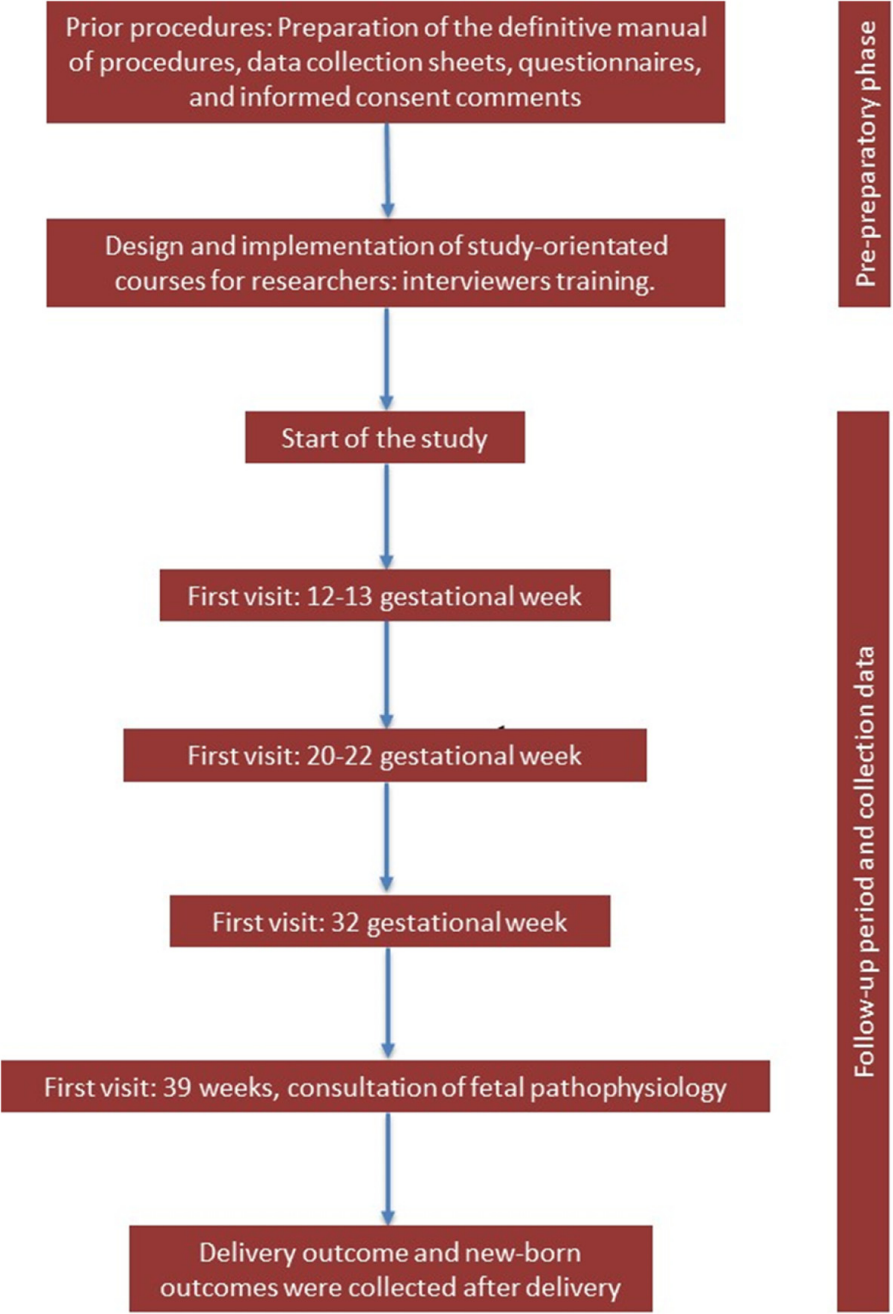
Time-line of study

This was a prospective follow-up study on a cohort of pregnant women aged 18 to 40 years from Cuenca city attending for three examinations that included ultrasound scans at the Virgen de la Luz Hospital of Cuenca during the three trimesters of pregnancy. Pregnant women were required to attend for a first trimester examination and were then followed up during their pregnancy periods. The recruitment of pregnant women continued for 18 months from the start of the study. Figure 1 shows the time-line of the study.

The study sample included the first group of consecutively recruited pregnant women who had been diagnosed in three primary care health centres in Cuenca city, Spain.

### Inclusion criteria

For participation in the study, pregnant women had to meet the following inclusion criteria: i) be 18 to 40 years, ii) gestational age ≤ than 13 weeks, and iii) have a single fetus. Also, women were required to attend during pregnancy for three monitoring ultrasounds, which were part of the pregnancy protocol of the obstetric department at the Virgen de la Luz Hospital in Cuenca.

### Exclusion criteria

Participants were excluded where they met any of the following criteria: i) had difficulties understanding the Spanish language, ii) resided outside of Cuenca city, iii) women who were not planning to give birth at Virgen de la Luz Hospital iv) had pre-existing conditions that prevented or limited the performance of physical activity at the time of recruitment (one or more contraindications for physical activity according to ACOG, pre-gestational diabetes, pre-pregnancy hypertension, more than one previous abortion), or, iv) had failed to sign the informed consent.

## Ethics and legal aspects

The study took into consideration the Declaration of Helsinki, as revised, as well as the norms of good clinical practice. Moreover, the study protocol had been approved by the Clinical Research Ethical Committee of the Virgen de la Luz Hospital.

Before inviting participants to sign the informed consent, information on the study goals and procedures was provided verbally. The participants were also invited to raise questions or doubts on any aspect of the study. Data confidentiality guarantees were provided to participants by the principal investigator.

## Study variables

### Pregnancy physical activity questionnaire

Physical activity was measured by the Pregnancy Physical Activity Questionnaire (PPAQ) [15], a self-administrated 32 item instrument that aims to measure in pregnant women the frequency and duration of the following physical activity related-behaviours: sedentariness, commuting, housework/caregiving, work-related tasking, and sports/exercise. A Spanish adapted version of the PPAQ was used, previously piloted in an earlier study on 10 pregnant women. On average, the completion of the questionnaire took 10 min.

The time reported in each activity was multiplied by its intensity to obtain a weekly average of energy expenditure (MET-h.wk21) and then summed to derive the weekly total activity. The weekly average energy expended was categorised according to four levels of physical activity intensity [16]: sedentary (<1.5 METs), light (1.5– < 3.0 METs), moderate (3.0–6.0 METs) or, vigorous (>6.0 METs).

*Physical activity behaviour:* a pilot questionnaire was used previously in an earlier study on 10 pregnant women. The final questionnaire collected data regarding:

i) Pre-pregnancy physical activity: profile of physical activity, number of days per week, type of exercise (walking, running, swimming, dancing, cycling), auto-reported physical activity level (low, moderate and high), and advice received by health professionals (Gynecologist and/or Midwife).
ii) Changes in physical activity behaviour during pregnancy (decreased, increased or no change in physical activity).
iii) Perceived barriers to physical activity during pregnancy (abortion fear, discomfort, lack of knowledge on benefits of physical activity during pregnancy, excess of tiredness, absence of exercise habits, insufficient time, weight gain…).
iv) Physical activity behaviour of their husbands/partners: time of exercise per week.
v) Health problems before pregnancy*.*

### Pregnancy symptoms inventory

The Pregnancy Symptoms Inventory (PSI) [17] was used to assess the nature and the frequency of the effects of pregnancy symptoms on women. It is a self-administered questionnaire that consists of 41 pregnancies related symptoms.

### Accelerometer measurements and sleep log

An accelerometer was used to objectively measure the level of physical activity over each pregnancy trimester. The women were instructed to wear the accelerometer 24 h (i.e. all the time) for 7 consecutive days and nights.

The Actigraph triaxial GT3X+ (Actigraph LLC, USA, 2009) accelerometer can measure activity counts in the vertical, horizontal right to left, and horizontal front to back, planes, and can generate a summative score of the three axes represented by vector magnitude [18–20]. The ActiGraph converts signals produced by a piezoelectric acceleration sensor in hertz. Samples are added in a sampling interval of time specified by the user, called an “epoch”. ActiGraph output is in the form of activity “counts”, where a count is equivalent to 16 m-g per second, and where g is equal to 9,825 ms^-2^, the acceleration of gravity. Activity counts are recorded to the internal memory of accelerometers by converting acceleration units over a given epoch [21]. Participants wore the accelerometer fastened with an elastic band to the right side of the waist for seven consecutive days to record habitual physical activity, except for bathing and performing activities in the water. All subjects were verbally instructed on how to use the accelerometer. The accelerometer was set to record physical activity data every minute. Sequences of 10 or more consecutive zero counts were considered non-wearing time and excluded from the analyses.

Data were downloaded using Actilife 6.0 software (Actigraph LLC, USA, 2013). The average total counts were defined as the mean vertical accelerometer’s output by 24 h period, reflecting the output without any categorisation according to intensity.

Inclusion criteria were a minimum of 4 days of recording, including at least 1 weekend day and at least 600 registered minutes per day. The main outcome variable from the activity monitor was the average intensity of physical activity (counts/minute), calculated with equal weighting given to each day (regardless of registered time per day). The intensity of physical activity was categorised according to the cut-off points proposed by Freedson [22]: sedentary (5724 counts min) and very vigorous (>9498 counts min). Moderate-vigorous activity was considered activity accumulated from all bouts lasting for at least one minute.

A sleep log was completed by the participants including information on the following variables: time of going to bed, time of waking up, total bedtime, napping behaviours, removal of the accelerometer (for bathing, swimming, convenience or comfort), and practised activities not capable of being detected by accelerometers such as static cycling.

### Nutritional behaviour

The Young Adolescents’ Nutrition Assessment on Computer (YANA-C) [23] was used to collect detailed dietary information from the pregnant women. The test consists of a single 24-h recall and is structured according to six meal occasions (breakfast, mid-morning snack, midday meal, afternoon snack, evening meal and evening snack) embedded within questions that take the participants through a range of sequential activities (i.e. when they woke up, what they did during the morning…). These additional questions provide a context, which helps participants recall what they have eaten. We conducted a previous pilot of the Spanish version of the YANA-C on 10 pregnant women.

### Other measurements

*Anthropometry and body composition:* Weight was measured twice (scale Seca ® 861; Seca, Germany, 2011) with the woman barefoot and in light clothing in pregnancy weeks 12, 20, 32, 39. Height was also measured twice, using a wall stadiometer (Seca® 222), with the woman barefoot, upright, and with the sagittal midline touching the back board. Body mass index (BMI) was calculated as weight in kg divided by the square of the height in meters. Self-reported weight was used to calculate pre-pregnancy BMI.

The gestational age was stabilised by the last menstruation period or by a first-trimester ultrasound measurement.

*Socio-demographic characteristics:* age (years), marital status (married, cohabiting, single not living with partner, others), country of origin, educational level (grammar school, secondary school, vocational training school or further education, bachelor level and advanced education), socioeconomic status (high, medium, low), employment status (homemaker, not employed, employed), and pre-pregnancy weight.

*Pregnancy data* including: gestational age (weeks + days), high-risk pregnancy related factors, smoking habits (number of cigarettes/day), medication, health problems (preterm delivery, hypertension, GDM (defined according the current ADA criteria) [24], intrauterine growth restriction, oligoamnios…), delivery preferences (analgesia during labour or low intervention birth), and, practice of special physical activity during pregnancy (pilates, swimming, stationary cycling…).

*Delivery data* including: gestational age (weeks + days), start of delivery (spontaneous/induced, reason for induction), type of delivery (normal/operative delivery/caesarean section), reasons for operative delivery/caesarean section/episiotomy, perineal lacerations, expulsion of the placenta (with or without the use of uterotonic agents/manual extraction of the placenta), duration of the first stage of labor (minutes), duration of the second stage of labour (minutes), and, duration of pushing efforts.

*Data for the new-born* including weight (grams), gender (male/female), Apgar score 1/5 min, type of resuscitation (I/II/III/IV), pH of umbilical cord blood (arterial or venous), intrauterus pH (if done), alterations of fetal heart rate (FHR) during labour, cord clamping (early/late), admission to neonates service, and effective breastfeeding in the first 2 h postpartum.

*Postpartum data* including expulsion of the placenta (with or without the use of uterotonic agents/manual extraction of the placenta), bleeding (physiological/moderate/severe), use of oxytocin, state of the perineum (without injuries/episiotomy/perineal laceration/hematoma/edema/pain/haemorrhoids), and postpartum complications.

Data on pregnancy, delivery, new-born and postpartum were collected from medical records through an ad hoc questionnaire by research members.

## Data collection time-points

Women attending for the first ultrasound examination (at 12–14 weeks gestation) were recruited consecutively throughout 18 months. Each participant was verbally informed about the study by a member of the research team and invited to participate and to sign the consent for participation. During the first visit, a member of the research team collected the following particulars: pre-pregnancy physical activity, anthropometric and body composition (weight, height and BMI), socio-demographic characteristics, and pregnancy data. In addition, information on nutritional behaviour was collected through a YANA-C test.

The women received portable accelerometers and were instructed verbally and in writing to wear the devices continuously for 7 days and nights and to remove them only for bathing or water sports. Finally, the women were given PPAQ and PSI tests and were also provided with sleep diaries.

During the second and third ultrasound scans (at 20–22 and 32 weeks, respectively), two new sets of data were collected (PPAQ, PSI, YANA-C, sleep diary, weight, and some obstetric characteristics that could have changed such as GDM or pregnancy hypertension). Also, accelerometers were provided to be worn for 7 days.

During the consultation of fetal pathophysiology (39 weeks), the women were weighed for the last time. After delivery, the following outcome measures were collected: i) pregnancy data (anthropometry and body composition, preeclampsia, GDM); ii) delivery data (type of delivery: normal/operative delivery/caesarean section); iii) postpartum data: bleeding (physiological/moderate/severe), and state of the perineum; and iv) data for the new-born (weight new-born, sex, Apgar score at 1-min and 5-min, type of resuscitation, intrauterus pH and pH of umbilical cord blood).

Data regarding pregnancy, delivery, new-born and postpartum were collected from medical records through an ad hoc questionnaire by research members.

## Statistical aspects

The sample size was calculated using Epidat 4.1 with a ratio of exposed/unexposed of 1. The outcome variable was GDM [24]; we assumed a GDM incidence rate of 14 % and 3 % [25] in the exposed and non-exposed groups, respectively. We also considered as exposed (sedentary) those women who did not meet the criteria of the American College of Sports Medicine for active people (-30 min/day of moderate-vigorous intensity physical activity every or almost every day until an accumulation of at least 120 min/week has been reached). A 5 % alpha error and 80 % statistical power were assumed. Following these premises, it was estimated that 194 pregnant women should be included in the study. To this figure 5 % was added for potential non-response (women who did not wish to participate in the study) and drop-outs.

Descriptive statistics with precision estimates were used to report the prevalence of each parameter using cross-sectional data. Mixed regression models [26] were estimated using each outcome variable as a dependent variable at the end of the study and controlling for baseline values. The results were expressed as absolute differences in changes in variables between the baseline and final measurements (IC of 95 %).

Differences were considered as statistically significant at *p <*0.05, and the analysis was performed using the 14 version of the STATA statistical package (StataCorp LP, USA, 2015). To the generalised lineal models, *glm* was used.

More detailed secondary analyses were conducted specific to each parameter, and non-parametric models were used where the data did not assume a normal distribution.

## Quality control

To ensure the quality of study data in order to maximise validity and reliability, several procedures were used:

- The same written documentation was provided to all participants.
- Interviewers were trained in the study’s characteristics and procedures in a 4 h session.
- Regular meetings for assessing the measurement process were held.

## Discussion

The present study aims to evaluate, under near real conditions, the influence of physical activity across the three trimesters of pregnancy on the following: i) pregnancy outcomes, ii) type of delivery, and iii) new-born outcomes.

It is known the positive effect of physical activity during pregnancy on maternal and fetal health. Moreover, the characteristics of physical activity (type, intensity and duration) remains controversial and studies have not identified the optimal features of physical activity associated with favourable pregnancy outcomes.

Recent studies have provided some evidence regarding the relationship between physical activity during pregnancy and its impact on delivery, mother and new-born outcomes. Physically active pregnant women, such as those performing at least 30 min of moderate physical activity during pregnancy, gained less weight during pregnancy [1], reduces new-born child weight (among normal values), [27] and experienced both improved cardiovascular fitness level and delivery outcomes [2].

Lastly, it will be essential to ascertain the nutritional behaviour of pregnant women. Prior research [28] found that 80 % of women who followed the recommendations of the US Institute of Medicine on Food and Nutrition [29] kept within the recommended weight gain during pregnancy and also reduced weight retention at two months postpartum. Further research is needed not only on women’s knowledge of, and practices in, nutrition, but on their dietary intake, and on their eating behaviours, in order to develop effective strategies to promote recommendations for healthy pregnancy and robust offspring.

Thus far, studies examining the effect of physical activity have focused either on athletes partaking in vigorous physical activity, or on people partaking in sedentary or low levels of physical activity, particularly women [2]. In Spain, there is a lack of studies quantifying the level of physical activity that pregnant women engage in, even though instruments to measure such physical activity are available [15]. Furthermore, there is a deficiency of studies assessing the validity of these instruments.

Most authors have recommended that questionnaires be used, not only to assess sports and recreational activities, but also to evaluate a full range of daily physical activities such as work-related physical activity, transportation and childcare. A similar approach has also been recommended for the purpose of assessing sedentary behaviour and sleep patterns as they affect pregnant women [30].

## Abbreviations

ACOG, American college of obstetricians and gynaecologists; BMI, body mass index; metabolic equivalent task (MET); GMD, gestational diabetes mellitus; PPAQ, pregnancy physical activity questionnaire; PSI, pregnancy symptoms inventory; YANA-C, young adolescents’ nutrition assessment on computer.
